# Neglected Very Long-Chain Hydrocarbons and the Incorporation of Body Surface Area Metrics Reveal Novel Perspectives for Cuticular Profile Analysis in Insects

**DOI:** 10.3390/insects13010083

**Published:** 2022-01-12

**Authors:** Marek Golian, Tanja Bien, Sebastian Schmelzle, Margy Alejandra Esparza-Mora, Dino Peter McMahon, Klaus Dreisewerd, Jan Buellesbach

**Affiliations:** 1 Institute for Evolution & Biodiversity, University of Münster, Hüfferstr. 1, D-48149 Münster, Germany; marek.golian@gmx.de; 2 Institute of Hygiene, University of Münster, Robert-Koch-Str. 41, D-48149 Münster, Germany; Tanja.Bien@ukmuenster.de (T.B.); klaus.dreisewerd@uni-muenster.de (K.D.); 3 Ecological Networks, Technical University of Darmstadt, Schnittspahnstr. 2, D-64287 Darmstadt, Germany; sebastianschmelzle@gmail.com; 4 Institute of Biology—Zoology, Free University of Berlin, Unter den Eichen 87, D-12205 Berlin, Germany; alejandra.esparza@fu-berlin.de (M.A.E.-M.); dino.mcmahon@fu-berlin.de (D.P.M.); 5 Department for Materials and Environment, BAM Federal Institute for Materials Research and Testing, Unter den Eichen 87, D-12205 Berlin, Germany

**Keywords:** cuticular hydrocarbons, Blattodea, GC-MS, Ag-LDI-MS, chemical ecology

## Abstract

**Simple Summary:**

The waxy layer covering the surface of most terrestrial insects is mainly composed of non-polar lipids termed cuticular hydrocarbons (CHCs). These have a long research history as important dual traits for both desiccation prevention and chemical communication. We analyzed CHC profiles of seven species of the insect order Blattodea (termites and cockroaches) with the most commonly applied chromatographic method, gas-chromatography coupled with mass spectrometry (GC-MS), and the more novel approach of silver-assisted laser desorption/ionization mass spectrometry (Ag-LDI-MS). Comparing these two analytical methods, we demonstrated that the conventional GC-MS approach does not provide enough information on the entire CHC profile range in the tested species. Ag-LDI-MS was able to detect very long-chain CHCs ranging up to C58, which remained undetected when solely relying on standard GC-MS analysis. Additionally, we measured the body surface areas of each tested species applying 3D scanning technology to assess their respective CHC amounts per mm^2^. When adjusting for body surface areas, proportional CHC quantity distributions shifted considerably between our studied species, suggesting the importance of including this factor when conducting quantitative CHC comparisons, particularly in insects that vary substantially in body size.

**Abstract:**

Most of our knowledge on insect cuticular hydrocarbons (CHCs) stems from analytical techniques based on gas-chromatography coupled with mass spectrometry (GC-MS). However, this method has its limits under standard conditions, particularly in detecting compounds beyond a chain length of around C40. Here, we compare the CHC chain length range detectable by GC-MS with the range assessed by silver-assisted laser desorption/ionization mass spectrometry (Ag-LDI-MS), a novel and rarely applied technique on insect CHCs, in seven species of the order Blattodea. For all tested species, we unveiled a considerable range of very long-chain CHCs up to C58, which are not detectable by standard GC-MS technology. This indicates that general studies on insect CHCs may frequently miss compounds in this range, and we encourage future studies to implement analytical techniques extending the conventionally accessed chain length range. Furthermore, we incorporate 3D scanned insect body surface areas as an additional factor for the comparative quantification of extracted CHC amounts between our study species. CHC quantity distributions differed considerably when adjusted for body surface areas as opposed to directly assessing extracted CHC amounts, suggesting that a more accurate evaluation of relative CHC quantities can be achieved by taking body surface areas into account.

## 1. Introduction

First described by Wigglesworth in 1933 as a complex fatty or waxy substance in the upper layer of the insect cuticle [1], the presence of non-polar hydrocarbons therein has subsequently been suggested [2,3], and later confirmed following the advent of gas-liquid chromatography [4]. Subsequently, from the late 1960s onwards, the development of gas-chromatography, coupled with mass spectrometry (GC-MS), became the standard for insect cuticular hydrocarbon analyses [5,6]. This led to the common assumption that insect CHC profiles mostly consist of hydrocarbons between 20 and 40 carbon atoms in length, as this is the range best assessed with GC-MS machines operating under standard conditions [7,8]. However, this methodology has its limitations, particularly for higher molecular weight compounds, as analytes need to be vaporized and separated on a capillary column for detection [8,9]. Accordingly, the boiling point of the analyzed compounds, which is directly correlated with the hydrocarbon chain length for CHCs, is the main criterion for the detectability by GC-MS technology [10,11]. Thus, if higher chain length CHCs are present on the insect cuticle, these might routinely be missed by overreliance on the standard analytical methods provided by GC-MS machines [9]. A few studies already assessed CHC compounds with chain lengths of up to C70 by other chromatographic and spectrometric methods, revealing a far larger repertoire of CHC compounds produced by insects than previously assumed [9,12,13]. Thus, the whole range of chain lengths actually present in insect CHC profiles is assessed rather rarely, which limits the scope of CHC studies towards the ones only accessible by conventional GC-MS techniques [8,9]. In the present study, we investigate the discrepancy between the insect CHC repertoire typically accessible by GC-MS and the range of very-long-chain CHCs beyond that. Specifically, we applied silver-assisted laser desorption/ionization mass spectrometry (Ag-LDI-MS) [12,13]. With this technique, adduct formation with the assistance of silver cations enables the detection of a broader range of analytes beyond chain length ranges typically assessed by standard GC-MS analyses [12].

We chose to compare representative species from the large and prominent insect order Blattodea, encompassing species that are highly variable in body size, sociality, and chemical communication mechanisms, all factors that have directly and indirectly been associated with CHC profile variation [14,15,16]. To cover the broad range of different levels of social organization within the Blattodea, we include both cockroach and termite species in our study, differing in life style, ecology and evolutionary history [17]. Our cockroach study species are *Blatta orientalis* (Blattodea: Blattidae) and *Blattella germanica* (Blattodea: Ectobiidae). The former is known as one of the most common cockroach pest species in temperate regions around the world [18,19,20], whereas the latter is well-established in CHC-based chemical ecological studies [21,22,23,24]. We further included five termite species differing in their levels of social organization, from species displaying low levels of social organization (*Kalotermes flavicollis* and *Neotermes castaneus*, Blattodea: Kalotermitidae) to phylogenetically more derived species with higher levels of social organization (*Reticulitermes flavipes* and *Coptotermes formosanus*, Blattodea: Rhinotermitidae), as well as the phylogenetically basal but socially advanced species *Mastotermes darwiniensis* (Blattodea: Mastotermitidae) [25,26,27,28].

With this diverse range of study species, we also attempt to address another common issue in conventional CHC analyses, namely the wide-spread lack of consideration of insect body size variations in proportion to CHC amounts (e.g., [29,30,31]). The body size of an organism is of paramount importance in determining how they interact with their physical environment. Organisms with a small body size, for example, possess a higher surface area to volume ratio than larger organisms, and this renders them particularly vulnerable to desiccation [32]. As CHCs play a major role in desiccation prevention, surface areas should be given more careful consideration when analyzing insect CHC profiles [8,33,34]. This becomes particularly important when conducting comparative studies on different insect species with large variations in body size. Thus, correcting CHC quantities for the respective body surface areas of the insects they were extracted from may enable a more accurate interpretation of proportional CHC quantity distributions. In our comparative study within the insect order Blattodea, body surface areas largely varied from 10 mm^2^ (*Reticulitermes flavipes*, *Coptotermes formosanus*) to over 500 mm^2^ (*Blatta orientalis*). Thus, we attempted to determine whether this considerable size, and thus surface area variation, influences relative CHC quantities conventionally quantified by GC-MS, as Ag-LDI-MS is not suited for accurate quantifications of total CHC content [12,13]. Therefore, we compared the uncorrected CHC amounts extracted from our seven representative Blattodea species with proportional CHC quantities per mm^2^ body surface area. We hypothesized that a more accurate and comprehensive assessment of CHC quantity distributions can be achieved when factoring in the respective insect body surface areas.

## 2. Materials and Methods

### 2.1. Tested Termite and Cockroach Species

Termite workers were retrieved from long-term colonies of *R. flavipes* (Rf)*, C. formosanus* (Cf)*, K. flavicollis* (Kf) and *N. castaneus* (Nc) maintained in the Federal Institute of Materials Research and Testing (BAM), Berlin. Termite colonies were kept in a darkened room at 26 °C and 84% humidity, except *M. darwiniensis* (Md) which was maintained at 28 °C and 83% humidity. All colonies were fed regularly with pre-decayed wood. The cockroaches *B. germanica* (Bg) and *B. orientalis* (Bo) were maintained in mixed open rearing boxes in 12-h light/dark cycles at 26 °C and 50% humidity, from the day of egg-laying until disposal of older adults. Cockroaches were reared on a mixture of 77.0% dog biscuit powder, 19.2% oat flakes, 3.8% brewer’s yeast and supplied with water ad libitum and weekly with apple and carrot slices. All cockroaches and termites were freeze-killed and stored at −20 °C until further analysis. 

### 2.2. GC-MS Analysis of Surface Extracts

To yield comparable amounts of extracts between cockroach and termite species, we had to adjust extraction volumes and pool smaller individuals. For this, we used 300 and 3000 µL MS pure hexane (UniSolv, Darmstadt, Germany) on single Bg and Bo individuals, respectively, and 100 µL on pools of three individuals per termite species for extraction. The following analytical procedures were then equalized to ensure comparability. Extractions were performed in glass vials (20 mL for cockroaches and 2 mL for termites, Agilent, Santa Clara, CA, USA) on an orbital shaker (IKA KS 130 Basic, Staufen, Germany) for 10 min. The extract derived from the termites was transferred to a 250 μL conical insert (Agilent, Santa Clara, CA, USA) and the extract derived from the cockroaches into a fresh 2 mL glass vial, where it was evaporated under a constant stream of gaseous carbon dioxide. Then, the dried extract was resuspended in 5 (termites), 100 (Bg) and 1000 (Bo) μL of a hexane solution containing 7.5 ng/μL dodecane (C12) as an internal standard, respectively. Three microliters of the resuspended extracts were then injected into a gas-chromatograph coupled with a tandem mass spectrometer (GC-MS/MS) (GC: 7890 B, Triple Quad: 7010 B; Agilent Technologies, Waldbronn, Germany) equipped with a fused silica column (DB-5 MS ultra inert; 30 m × 250 μm × 0.25 μm; Agilent J&W GC columns, Santa Clara, CA, USA) in splitless mode at a temperature of 300 °C with helium used as a carrier gas under constant flow of 2.25 mL/min. The temperature program started at 60 °C held for 5 min, increasing 20 °C per minute up to 200 °C and then increasing 3 °C per minute to the final temperature of 325 °C, held for 5 min. 

Sample sizes for the analyzed CHC extracts were Bg: 9 (5 females, 4 males), Bo: 10 (6 females, 4 males), Cf: 5, Kf: 4, Md: 4, Nc: 5 and Rf: 5 (all workers). CHC peak detection, integration, quantification and identification were all carried out with Quantitative Analysis MassHunter Workstation Software (Version B.09.00/Build 9.0.647.0, Agilent Technologies, Santa Clara, CA, USA). The pre-defined integrator Agile 2 was used for the peak integration algorithm to allow for maximum flexibility, and quantification was carried out over total ion count (TIC). All peaks were then additionally checked for correct integration and quantification, and where necessary, re-integrated manually. CHC compound identification was then carried out based on their characteristic diagnostic ions and retention indices as indicated in detail in supplementary Table S1. Relative CHC ratios were obtained by standardizing the individually obtained peak areas for each CHC compound by the overall sum of all peak areas per individual. Absolute CHC quantities (in ng) were obtained by standardizing all peak areas of the identified CHC compounds according to the concentration of the internal C12 standard. In the case of pooled termites, those were additionally divided by three to obtain mean total CHC amounts per single individual.

### 2.3. Ag-LDI-MS Analysis of Surface Extracts

Preparation of etched silver substrates was carried out as described previously [12,13]. For the CHC extracts, pools of three termites were placed in 2 mL and single cockroaches in 20 mL glass vials. Termites were extracted with 100 µL of chromatography grade heptane (Merck, Darmstadt, Germany). For the cockroaches, we used 300 µL of heptane for *B. germanica* and 3000 µL of heptane for *B. orientalis*. The extraction took place for 10 min on a rotary shaker (IKA KS 130 Basic, Staufen, Germany) before the insects were removed. The extracts were dried under a constant flow of gaseous carbon dioxide and resuspended in 5 (termites), 100 (Bg) and 1000 (Bo) μL of heptane; 0.5 µL of the resuspended extract was then spotted onto the center of the etched sliver plates. The employed mass spectrometer was a timsTOF fleX MALDI-2 prototype (Bruker Daltonik, Bremen, Germany) providing a mass resolving power of about 40,000 (fwhm). The instrument was equipped with a SmartBeam 3D laser of 355 nm wavelength and 10 kHz pulse repetition rate. It was operated at an elevated pressure of about 3 mbar of N_2_ in the dual funnel ion source. All measurements were acquired in the positive ion mode in a mass range of *m/z* 300–1500. The laser power was set to 100%, and a total of 10,000 shots were applied to random positions on the silver plate, with 500 shots per position each. The sum spectra underwent lock-mass correction and automated peak picking in DataAnalysis (Version 5.3, Bruker Daltonik, Bremen, Germany). For this, a mass list with the monoisotopic ^107^Ag-adducts of possible saturated and unsaturated hydrocarbons with carbon numbers from C25–C58 and carbon-carbon double bonds from 0–3 was used to identify all signals with a signal-to-noise ratio set to >2 by exact mass (mass window: 0.005 Da). Sample sizes for the analyzed etched silver substrate extracts were Bg: 6 (3 females, 3 males), Bo: 5 (3 females, 2 males), Cf: 4, Kf: 6, Md: 5, Nc: 6 and Rf: 6 (all workers). The signal intensities of all signals were extracted and further processed to calculate the CHC signal-intensity composition in the respective chain length range. Note that no distinctions could be made between *n*-alkanes and methyl-branched alkanes due to the lack of diagnostic ions for further identification. Therefore, the distinction between chain length and the number of C atoms cannot be made as with GC-MS assessed CHC compounds. All detected signal intensities and the translation key to the respective CHC compound group (saturated, mono-, di-, and tri-unsaturated) are given in the supplementary Table S2. 

For both analytical methods, we restricted ourselves to termite workers to focus on general chemical profiles and avoid any caste-related variations. Similarly, we pooled the sexes of both respective cockroach species to focus on representative species-specific chemical profiles, with less emphasis on the more subtle sex-specific differences [35]. We aimed extraction procedures for both of our analytical methods to specifically target surface CHC compounds through short extraction times (10 min) and the use of two organic, non-polar solvents (hexane/heptane), which have been demonstrated to yield interchangeable, almost identical extraction results [36,37]. Note that heptane is less toxic than hexane and therefore preferable for performing Ag-LDI-MS, which requires manual spotting of the resuspended extracts. Furthermore, Ag-LDI-MS has been demonstrated to be similarly specific for surface compounds as dUV-LDI-MS, a technique applying a UV-Laser beam directly on insect surfaces to exclusively analyze cuticular compounds [12]. This reinforces that both of our methodologies exclusively yielded compounds from the insects’ epicuticular surfaces.

### 2.4. Surface Area Measurements

Extended depth of field (EDOF) images of the samples were recorded with the Darmstadt Insect Scanner DISC3D [38] with 398 imaged poses ranging from −70° to +70° for each sample. For the termites, a Schneider Componon 12 50 mm/2.8 lens in reverse position (Jos. Schneider Optische Werke GmbH, Bad Kreuznach, Germany), a Basler aca4112–20 uc 20 Mpx camera (Basler AG, Ahrensburg, Germany) and 96.5 mm extension tubes in between the former two were used. This resulted in a reproduction scale of 1.41 with a field of view of 10 mm. EDOF images of the cockroaches were recorded using the same camera, but equipped with a Schneider Componon 12 28 mm/2.8 lens (Jos. Schneider Optische Werke GmbH, Bad Kreuznach, Germany) and 20 mm extension tubes in between the former two. This resulted in a reproduction scale of 0.42 with a field of view of 34 mm. The textured 3D surface models were then generated based on the EDOF images and measured using the photogrammetry software Agisoft Metashape Pro 1.6.5 (Agisoft LLC, St. Petersburg, Russia). One representative 3D scan was obtained per tested species, which has been established as reliable and sufficient standard procedure [32,38]. The interactive 3D images of each tested species are accessible in Supplementary Figure S1.

## 3. Results

### 3.1. CHC Chain Length Distributions Incorporating Very-Long-Chain CHCs

**Figure 1:**
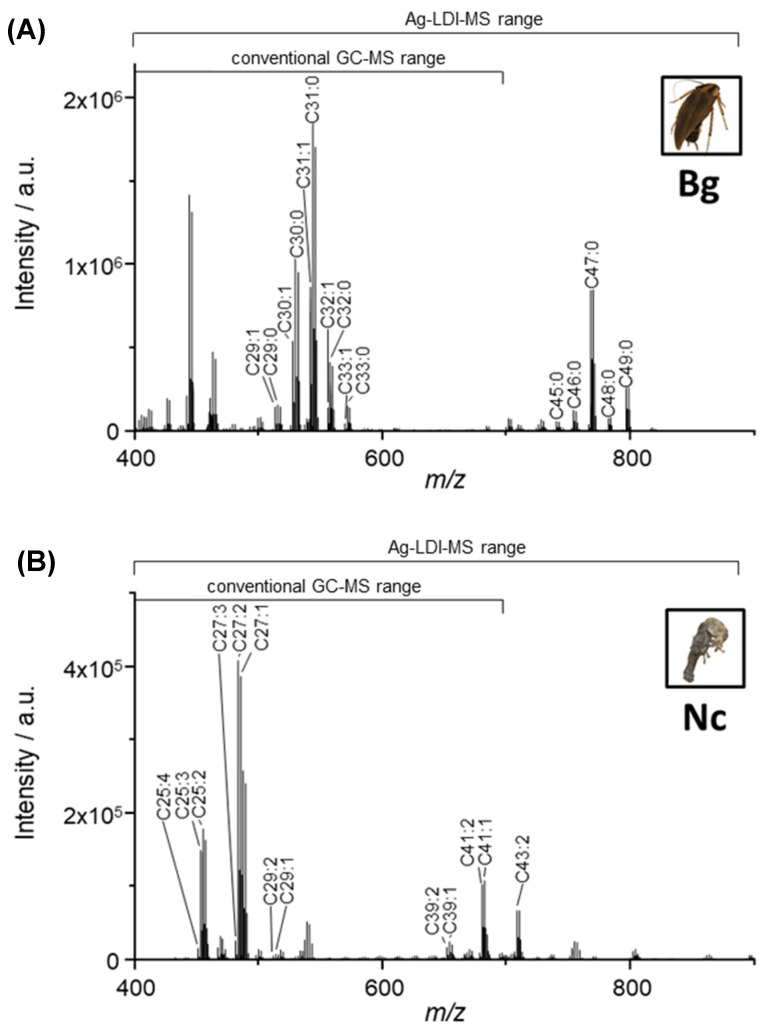
Ag-LDI mass spectra of heptane extracts from (**A**) a single *B. germanica* (Bg) and (**B**) a pool of three *N. castaneus* (Nc). All CHCs are detected as [M+^107^Ag]^+^/ [M+^109^Ag]^+^ doublets. Major ion signals are annotated with their putative identities as [M+^107^Ag]^+^-adducts based on the exact mass measurement with a mass accuracy of <5 ppm. The chain length range simultaneously accessible by conventional GC-MS is indicated to contrast it with the wider chain length range detectable by Ag-LDI-MS. Insect images have been obtained from their DISC3D scans (see Figure S1). Ag-LDI mass spectra of the remaining species are shown in Supplementary Figure S2, comparative GC-MS chromatograms in Figure S3.

**Figure 2:**
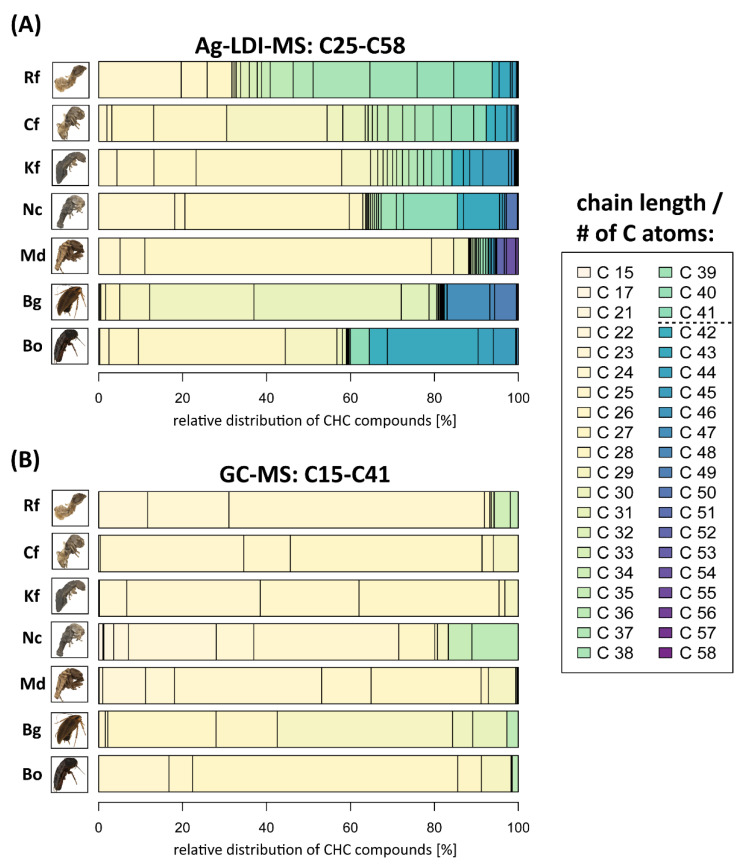
Relative distributions (in percentages) of CHC compounds in our seven studied Blattodea species grouped by chain length/number of C atoms. (**A**) C25–C58 range, obtained by the signal intensities measured with Ag-LDI-MS. (**B**) C15–C41 range, obtained by CHC quantities measured by standard GC-MS methodology. Note that signal intensities in terms of quantification in both methods are not directly comparable, hence only relative distribution ranges are given. Ranges in chain lengths/number of C atoms are indicated by a color gradient (light yellow to dark violet for lowest to highest chain length/number of C atoms, respectively). A dotted line between C41 and C42 in the legend indicates the chain length/number of C atoms detection limit for standard GC-MS methodology. Insect images have been obtained from their DISC3D scans (Figure S1). Rf: *Reticulitermes flavipes*, Cf: *Coptotermes formosanus*, Kf: *Kalotermes flavicollis*, Md: *Mastotermes darwiniensis*, Nc: *Neotermes castaneus*, Bo: *Blatta orientalis* and Bg: *Blattella germanica*.

Applying Ag-LDI-MS to the extracts, we were able to detect saturated and unsaturated CHCs in a broad range of chain lengths from 25 to 58 carbon atoms (Figure 1 and Figure 2). Figure 1 shows representative Ag-LDI-MS spectra of a cockroach (*B. germanica*, A) and a termite (*N. castaneus*, B), respectively. Both spectra display unique CHC profiles, with the cockroach mostly showing saturated and the termite mostly showing singly and multiply unsaturated CHCs (see also Table S1 and S2). However, a characteristic feature that both spectra share is the presence of two most conspicuous groups of CHCs with the respective highest intensities – one in a lower chain length range (approx. C25–C33) and one in the higher chain length range (approx. C39–C50). 

Figure 2 directly compares relative CHC compound distributions according to chain length ranges/number of C atoms accessible by Ag-LDI-MS (A) and by standard GC-MS methodology (B). In all our seven tested Blattodea species, chain lengths beyond C41 have been detected by Ag-LDI-MS that remained completely undetected in our GC-MS analysis. Chain lengths beyond C50 were detected for the cockroach *B. germanica* and the termites *K. flavicollis* and *M. darwiniensis*. Note that the relative proportions of CHC chain length/number of C atom distributions in the range overlapping between the two applied techniques differ drastically. However, a direct quantitative comparison cannot be made due to the differences in the ionization mechanisms between the GC-MS and Ag-LDI-MS methodologies. Therefore, only the relative distributions are given to adequately visualize the vast differences in accessed chain length ranges and their respective distributions.

### 3.2. Assessment of CHC Amounts in Relation to Body Surface Areas

**Figure 3:**
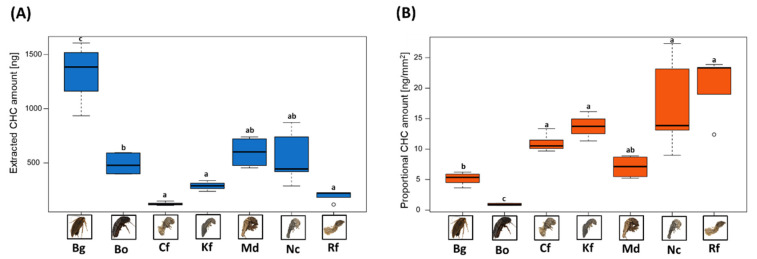
(**A**) Total extracted CHC amount (assessed and quantified by GC-MS) per individuum of our seven tested Blattodea species in ng. (**B**) Proportional CHC amount per individual in ng per mm^2^ body surface area. Means and standard deviations are given, statistical comparisons have been performed with Hommel corrected Mann-Witney U tests, significant differences are indicated by different letters. Species abbreviations and insect images are depicted as in Figure 2.

Restricting ourselves to GC-MS-assessed CHC quantities (as Ag-LDI-MS is less suited for exact compound quantifications [13]), we extracted the highest average CHC amount from *B. germanica* with over 1300 ng, more than twice as much as the second-highest average amounts of CHCs (around 600 ng) obtained from the termites *M. darwiniensis* and *N.* *castaneus* (Figure 3A). With around 120 ng, the termite *C. formosanus* yielded the lowest average CHC amount from our tested species. Conversely, when comparing CHC amounts proportional to the respective body surface areas of our study species (Table 1), different overall patterns emerge (Figure 3B). 

The two cockroach species which have the highest body surface areas also display the lowest proportional CHC amounts (around 0.9 and 5 ng/mm^2^ for B. orientalis and B. germanica, respectively). This is particularly interesting for B. germanica in direct comparison to the highest total CHC amount this species yielded. The species with the lowest body surface area displays the highest proportional CHC amount (R. flavipes). Among the tested termite species, M. darwiniensis displays the lowest proportional CHC amount, while yielding the highest average CHC amount. 

## 4. Discussion

Our study highlights the differences between the CHC profile range accessed by commonly applied GC-MS methodology and the considerable range of very long-chain CHC compounds detectable when utilizing more novel analytical techniques, in this case silver-assisted laser desorption/ionization mass spectrometry (Ag-LDI-MS). This demonstrates that large portions of CHC profiles may routinely be overlooked in chemical ecological studies solely relying on standard GC-MS methodology [8,31]. This constitutes a potential issue when considering how ubiquitously it is assumed that the whole range of CHCs is covered in most of these studies, despite them mostly lacking the possibility to access CHCs beyond C41 (e.g., [39,40,41]). Particularly in studies contemplating CHC profiles as chemical signaling cues (e.g., [42,43,44]), only assessing incomplete CHC profiles might potentially miss important information. In the majority of cases, CHC profiles are considered as a whole for conveying differential chemical information through cumulative qualitative and quantitative compound differences [45,46,47,48,49]. Conversely, the exact compounds actually encoding chemical information within CHC profiles have remained largely elusive except in a few case studies [50,51,52,53]. Similarly, the exact mechanisms of CHC perception, most importantly which particular compounds are perceivable and elicit a physiological and/or behavioral response, are mostly unknown as well [54,55,56]. Due to these largely elusive factors, the wide-spread neglect of compounds beyond the chain length range conventionally accessed through GC-MS is all the more surprising. Since CHC profile differences associated with chemical signaling mechanisms might as well extend to these rarely assessed very long-chain CHC compounds, it appears logical to at least acknowledge and consider them in future chemical ecological studies. This might prove particularly useful if these very long-chain CHC compounds constitute a non-negligible portion of the overall CHC profile, which is at least the case for most of our studied species within the order Blattodea (compare Figure 1 and Figure 2). Cvacka et al. [9] obtained similar results by applying matrix-assisted laser desorption/ionization (MALDI) time-of-flight (TOF) mass spectrometry for several termite species of the genera *Reticulitermes*, *Coptotermes* and *Neotermes*, including the ones studied here, confirming occurrence and prominence of very long chain CHCs in these taxa. 

However, it will remain a particular challenge in future studies to identify the exact structure of very long-chain CHCs without the added benefit of chromatographic separation of individual CHCs and their diagnostic ions. Due to the lacking separation in Ag-LDI-MS, all isomeric ions appear as one peak in the mass spectra. The precise compound identities of the very long-chain hydrocarbons can therefore not be unambiguously determined, particularly methyl-branch positions, or even the distinction between methyl-branched and straight-chain alkanes. In this respect, it might be helpful to incorporate high-temperature GC-MS technology and draw comparisons to the results obtained by Ag-LDI-MS. Most GC-MS studies on insect CHCs including ours are conducted on fused silica columns with a polyimide outer coating to enhance the efficiency in detecting and chromatographically separating non-polar compounds, with a temperature limit of around 325 °C [57,58]. Specifically fabricated high temperature columns being able to operate in temperatures as high as 400 °C also enable the detection of higher chain length CHCs beyond C41 [Schmitt & Karpati, pers. comm.], albeit not as far-reaching as the Ag-LDI-MS technique applied in this study or other techniques such as MALDI-TOF MS [9,58,59]. Nevertheless, future studies should attempt to integrate high temperature GC-MS techniques to unambiguously identify CHCs in the very long chain length range, which will be indispensable to advance our knowledge of the identity and functionality of these compounds.

For unsaturated CHCs, however, Ag-LDI-MS appears to be more sensitive than GC-MS, as evidenced by the detection of several unsaturated compounds within the overlapping chain length range not detectable by GC-MS (compare Tables S1 and S2). While GC-MS is relatively sensitive for saturated, shorter chain CHCs, the sensitivity of the ionization process in Ag-LDI mostly depends on the stability of the Ag-adduct and the pressure in the ion-source. This results in a higher sensitivity for unsaturated as compared to saturated compounds, since the respective Ag-adducts are more stable when double bonds are present [60,61]. Consequently, (poly) unsaturated CHCs can be detected in the Ag-LDI spectra that are hardly detectable in GC-MS analyses. Shorter chain CHCs, on the other hand, generally vaporize under the vacuum conditions in the ion source and are more likely missed by Ag-LDI-MS, particularly when present in trace amounts. This is noticeable when comparing relative compound distributions in both the differential and overlapping chain length ranges assessed by both techniques (Figure 2). This makes a direct comparison of both methods difficult in addition to the fact that Ag-LDI is less suited for absolute quantifications [12,13]. 

Another important but often-neglected factor in CHC studies is the consideration of different insect body sizes when assessing total CHC amounts. Insect body sizes translate to their respective surface areas, which in turn can influence total CHC quantities and distributions across the insect body [32,62]. Moreover, the primary function of CHCs is the prevention of desiccation, where the insects’ body surface also plays a major role [8,33,34]. Nevertheless, insect body surface areas are rarely considered in CHC studies, particularly when comparing overall CHC amounts (e.g., [29,30,31]). Therefore, we calculated proportional CHC amounts, factoring in the largely variant body surface areas of our studied Blattodea species, and compared them to unadjusted extracted CHC amounts. Our results highlight different proportional distributions between the two assessment methods, indicating that extracted CHC amounts alone may not always reflect accurate CHC quantity ratios without considering body surface areas. Since our overall sample size is rather limited to draw more general conclusions, it may be useful to factor in body surface areas in future studies investigating overall CHC quantities, especially when comparing insects with very divergent body sizes. This might also prove particularly useful in studies assessing differences in desiccation resistance, as body surface areas also likely constitute an important additional factor here. Furthermore, as our study clearly demonstrates, GC-MS assessed CHC quantities do not always include the entire CHC compound range present on the insect cuticle. This potentially further restricts the validity of quantitative comparisons between solely GC-MS assessed CHC amounts. Clearly, reliable quantification techniques of compounds beyond the GC-MS accessible range will be necessary for a holistic assessment of the whole CHC repertoire. However, other factors such as cuticle thickness, degree of sclerotization or morphological structures such as hairs also potentially influence both overall CHC amounts, as well as body surface areas, and should also be considered in future studies [63,64,65].

## 5. Conclusions

We report a considerable, usually neglected portion of very long-chain CHCs in chemical profiles from a variety of termite and cockroach species that are not typically detected by conventional GC-MS methods. This suggests that insect CHC profiles are rarely assessed in their entirety when solely relying on this technique. Therefore, we encourage the incorporation of analytical techniques capable of detecting very long-chain CHCs in future chemical ecological studies, which so far apparently mostly focused on only a subset of all the compounds potentially present on insect surface profiles. Furthermore, we demonstrate how the inclusion of insect body surface areas when measuring CHC amounts can shift the perspective on CHC quantity distributions, particularly when comparing insects with large body size variations. Overall, our results highlight two neglected areas in most commonly conducted studies on insect CHC profiles, and their incorporation in future research bears the potential for more comprehensive and accurate assessments of CHC variations, as well as CHC-based physiological and behavioral mechanisms.

## Figures and Tables

**Table 1:** Body surface area measurements in mm^2^ based on one representative DISC 3D scan for each of the seven tested Blattodea species. Species abbreviations as in Figure 2, interactive 3D images of each tested species are accessible in Figure S1.

Species	Surface [mm^2^]
Rf	9.58
Cf	11.07
Kf	20.97
Md	86.94
Nc	31.99
Bo	536.97
Bg	258.13

## Data Availability

All data underlying the presented study are available at the Dryad data repository under https://doi.org/10.6078/D1QT36 (9 January 2022).

## Supplementary Materials

The following supporting information can be downloaded at: https://www.mdpi.com/article/10.3390/insects13010083/s1. Relative abundances and CHC compound identifications assessed by GC-MS (Table S1), signal intensities and translation into CHC compound groups assessed by Ag-LDI-MS (Table S2), representative GC-MS chromatograms of all tested Blattodea species (Figure S2), representative DISC3D scans of all tested Blattodea species (Figure S1, top to bottom: *Blatta orientalis*, *Blattella germanica, Coptotermes formosanus*, *Kalotermes flavicollis*, *Mastotermes darwiniensis*, *Neotermes castaneus*, *Reticulitermes flavipes*), representative Ag-LDI mass spectra of the tested Blattodea species not shown in Figure 1 (Figure S2), representative GC-MS chromatograms of all tested Blattodea species (Figure S3, for species abbreviations see Figure 2).
