# Supplementary material for: Neglected Very Long-Chain Hydrocarbons and the Incorporation of Body Surface Area Metrics Reveal Novel Perspectives for Cuticular Profile Analysis in Insects
Source: Insects. 2022 Jan 12;13(1):83. doi: 10.3390/insects13010083 (PMC8778109; doi:10.3390/insects13010083)
Supplement: Supplementary file 1 [file insects-13-00083-s001.zip › Fig-S2.pdf]

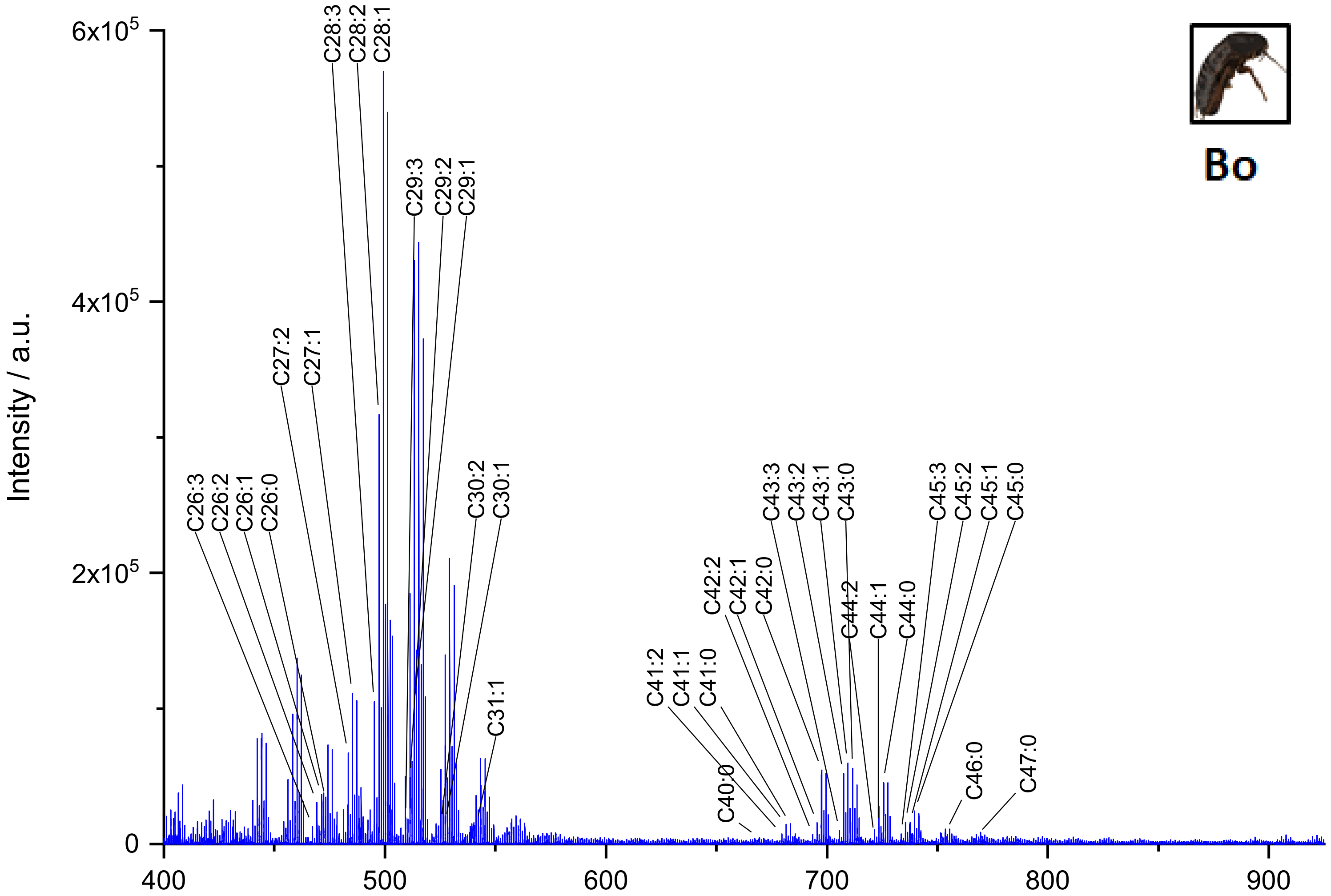

Ag-LDI mass spectra of a heptane extract from a single *B. orientalis* (Bo). All CHCs are detected as M+107Ag]$^+$/ [M+109Ag]$^+$ doublets. All signals in this spectra are annotated with their putative identities as [M+107Ag]$^+$-adducts based on the exact mass measurement with a mass accuracy of < 5 ppm. Please note, that signal intensities the presence of individual signals can vary between single insects.

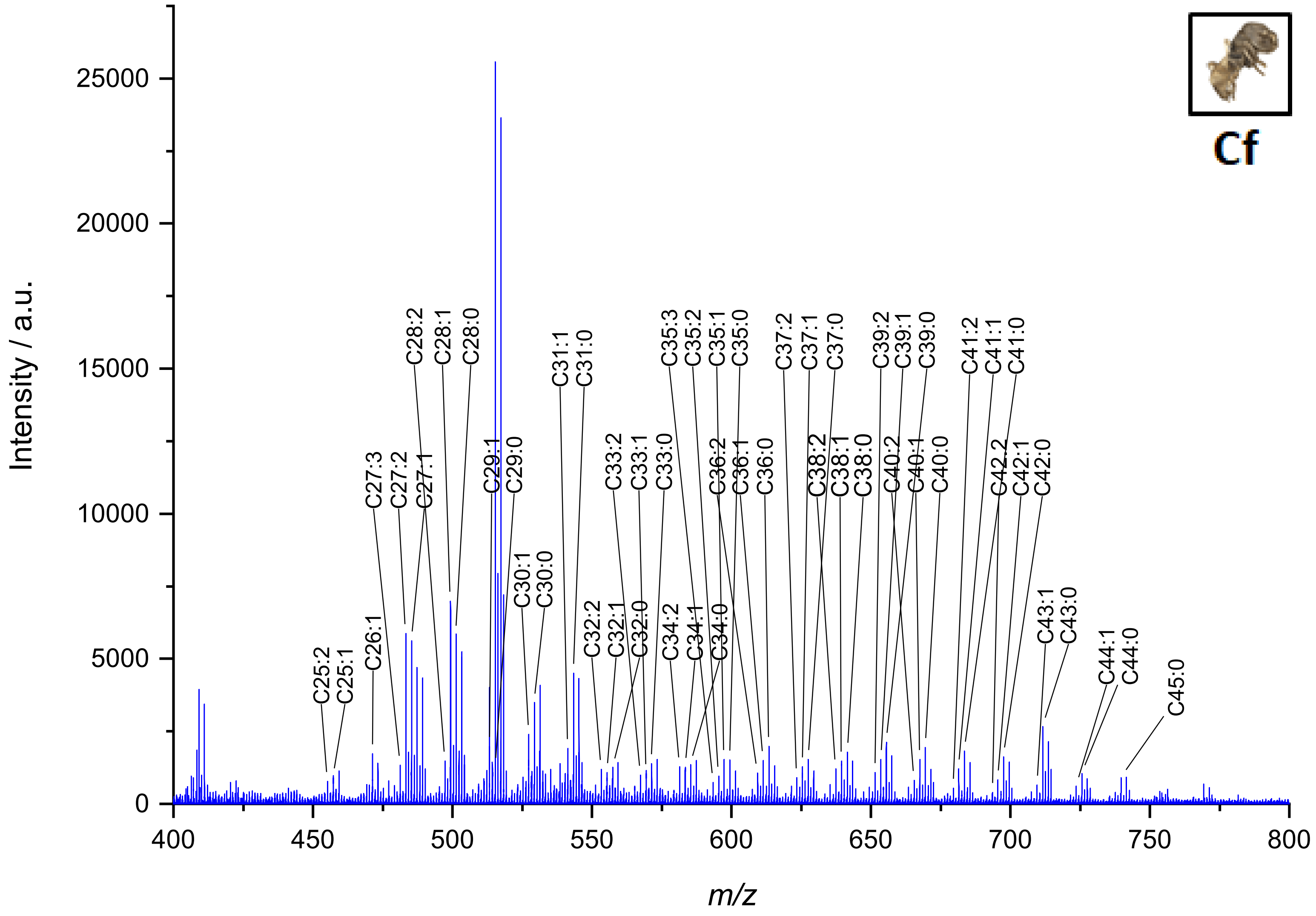

Ag-LDI mass spectra of a heptane extract from a pool of three *C. formosanus* (Cf). All CHCs are detected as M+107Ag]⁺/ [M+109Ag]⁺ doublets. All signals in this spectra are annotated with their putative identities as [M+107Ag]⁺-adducts based on the exact mass measurement with a mass accuracy of < 5 ppm. Please note, that signal intensities the presence of individual signals can vary between single insects.

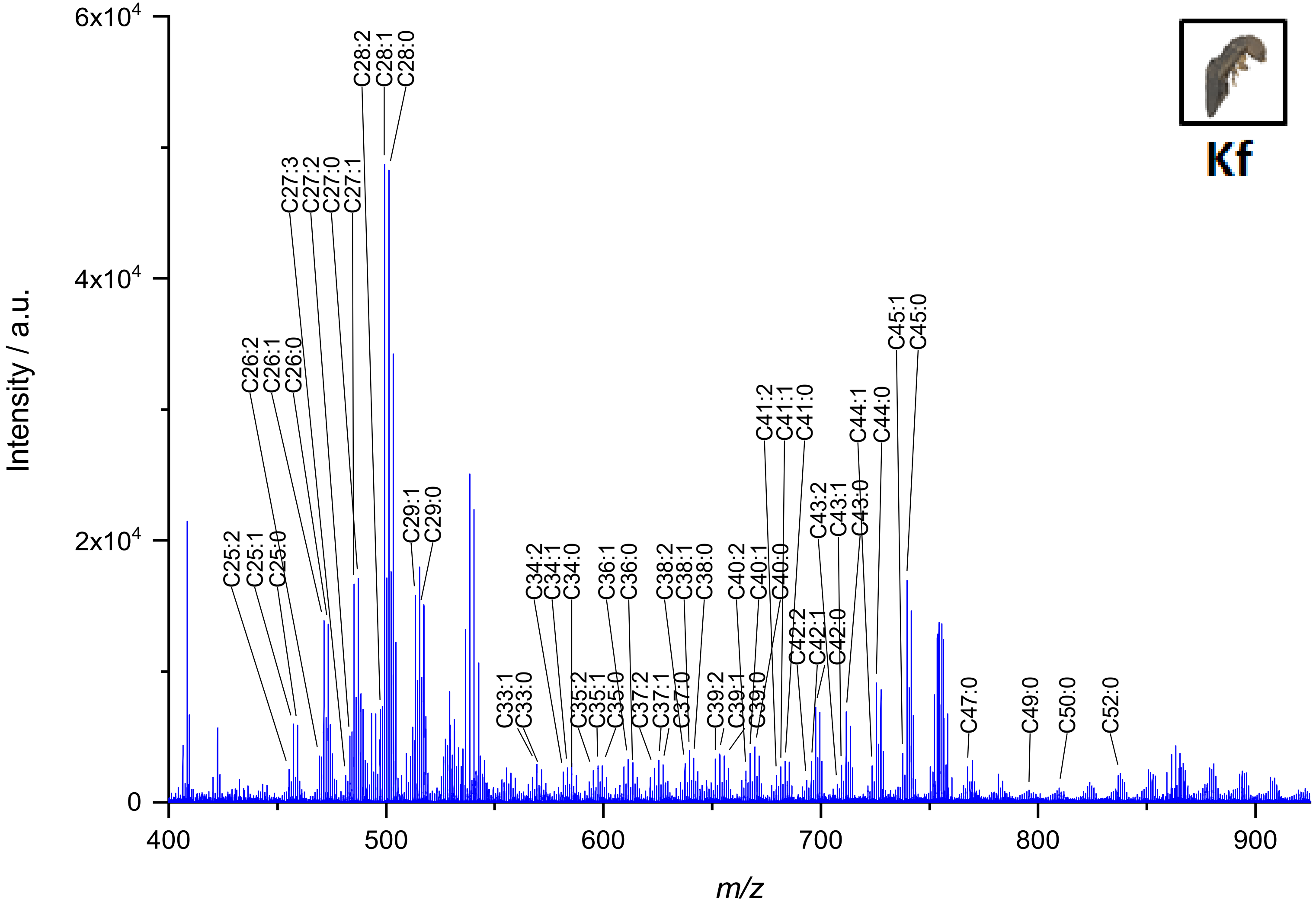

Ag-LDI mass spectra of a heptane extract from a pool of three *K. flavicollis* (Kf). All CHCs are detected as M+107Ag]$^+$/ [M+109Ag]$^+$ doublets. All signals in this spectra are annotated with their putative identities as [M+107Ag]$^+$-adducts based on the exact mass measurement with a mass accuracy of < 5 ppm. Please note, that signal intensities the presence of individual signals can vary between single insects.

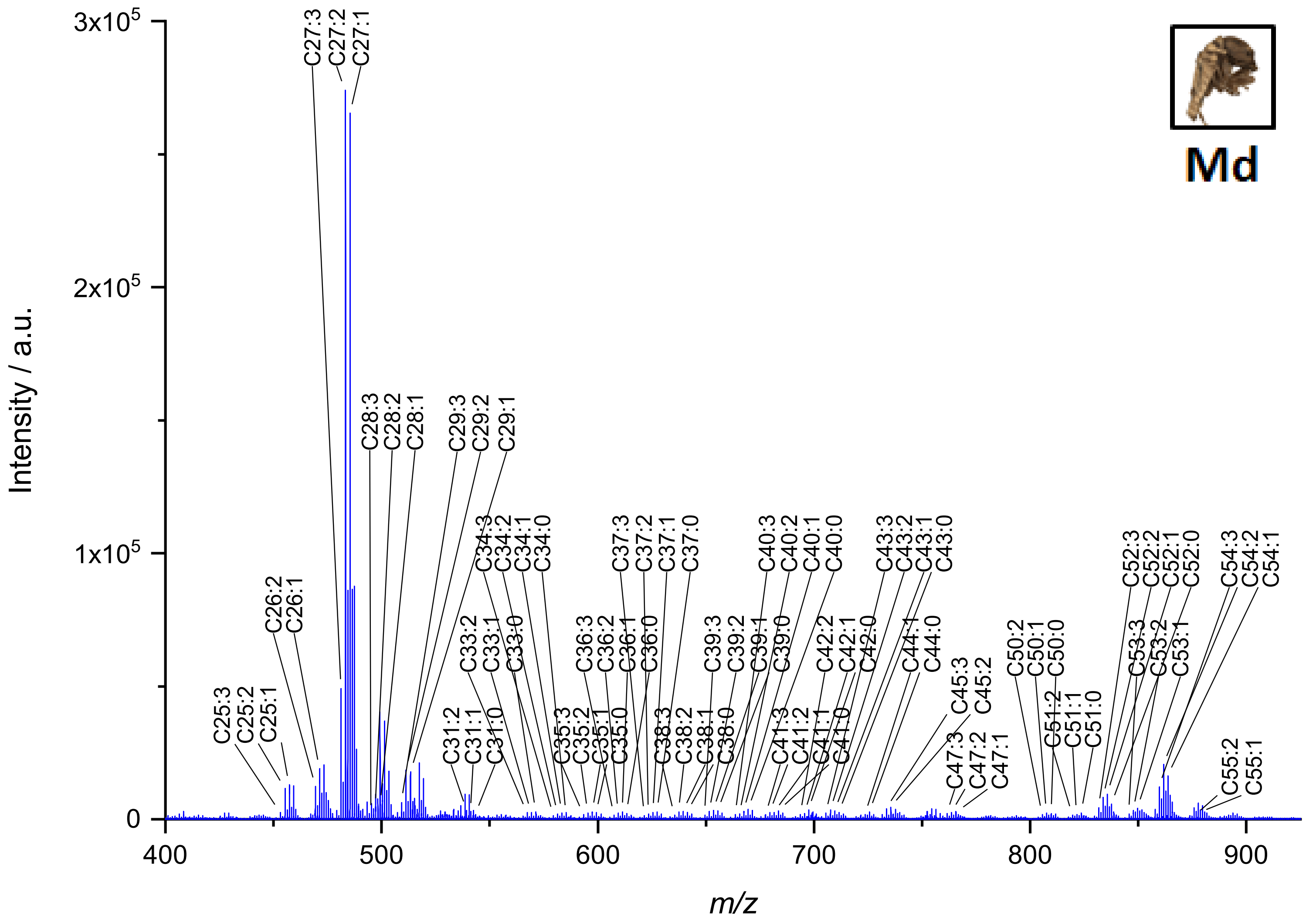

Figure S-1: Ag-LDI mass spectra of a heptane extract from a pool of three *M. darwiniensis* (Md). All CHCs are detected as M+107Ag]+/[M+109Ag]+ doublets. All signals in this spectra are annotated with their putative identities as [M+107Ag]+-adducts based on the exact mass measurement with a mass accuracy of < 5 ppm. Please note, that signal intensities the presence of individual signals can vary between single insects.

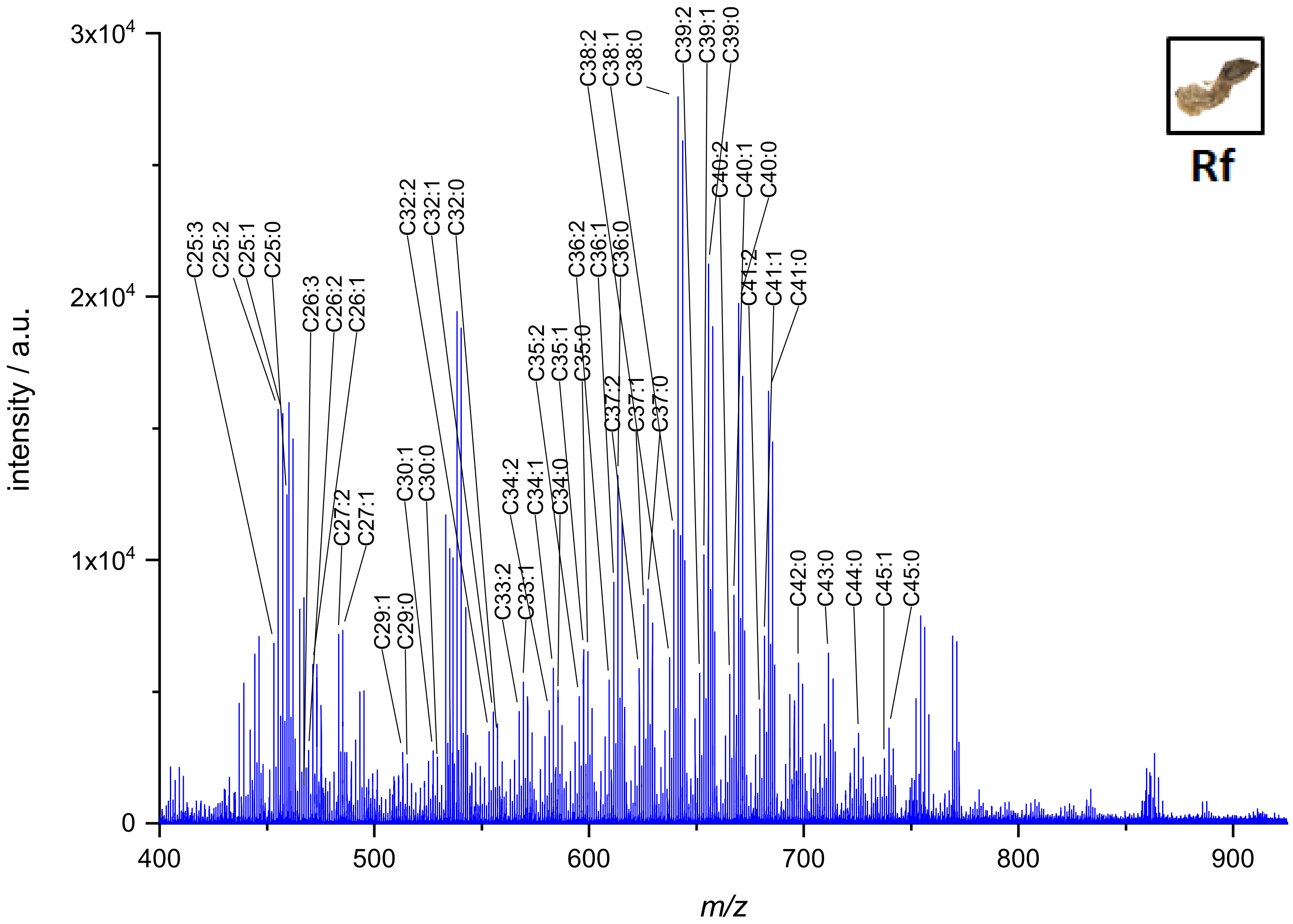

Ag-LDI mass spectra of a heptane extract from a pool of three *R. flavipes* (Rf). All CHCs are detected as M+107Ag]⁺/ [M+109Ag]⁺ doublets. All signals in this spectra are annotated with their putative identities as [M+107Ag]⁺-adducts based on the exact mass measurement with a mass accuracy of < 5 ppm. Please note, that signal intensities the presence of individual signals can vary between single insects.
