# Supplementary material for: Neglected Very Long-Chain Hydrocarbons and the Incorporation of Body Surface Area Metrics Reveal Novel Perspectives for Cuticular Profile Analysis in Insects
Source: Insects. 2022 Jan 12;13(1):83. doi: 10.3390/insects13010083 (PMC8778109; doi:10.3390/insects13010083)
Supplement: Supplementary file 1 [file insects-13-00083-s001.zip › Tab-S1.pdf]

Table S1: Retention indices (RI), CHC compound identifications and the respective relative quantities as well as standard deviations (in %) for all tested cockroach and termite species. Non-detectable amounts of the respective compounds are indicated by hyphens. Where compound identifications were ambiguous due to multiple possible methyl branch positions based on the detected ion pairs, all possible compound configurations are given. Retention indices were calculated according to the position of detected *n*-alkanes in our samples and, where not available, with a C21-40 *n*-alkane standard run under the same conditions. Please note that for compounds beyond C40, the *n*-alkane positions had to be extrapolated according to the average distances between *n*-alkanes.

RI	Compound ID	Diagnostic ions	Bg	Bo	Nc	Kf	Md	Cf	Rf
1500	n-C15	212	-	-	1.04 ± 0.05	-	-	-	-
1700	n-C17	240	-	-	0.06 ± 0.03	-	0.01	-	-
1738	3-MeC17	239; 56; 224	-	-	0.14 ± 0.07	-	-	-	-
2107	n-C21	296	-	-	2.33 ± 0.26	0.09 ± 0.04	0.1 ± 0.03	-	-
2207	n-C22	310	-	-	2.49 ± 0.14	-	0.82 ± 0.24	-	0.06 ± 0.03
2270	4-MeC22	309; 70; 280	-	-	1.03 ± 0.15	-	-	-	-
2280	C23-ene1	322	-	-	-	-	0.21 ± 0.06	-	1.13 ± 0.46
2287	C23-ene2	322	-	-	-	-	0.06 ± 0.02	-	0.07 ± 0.04
2310	n-C23	324	0.07 ± 0.07	-	13.48 ± 1.12	0.07 ± 0.02	7.57 ± 0.71	-	7.97 ± 1.38
2343	9-; 11-;13-MeC23	323; 140; 224; 168; 196;	0.01 ± 0.02	-	0.83 ± 0.22	0.01 ± 0.01	0.56 ± 0.18	-	0.39 ± 0.18
2349	7-MeC23	323; 112; 252	-	-	-	-	0.09 ± 0.03	-	-
2371	4-MeC23	323; 70; 294	-	-	3.45 ± 0.2	-	0.89 ± 0.26	-	1.46 ± 0.56
2379	7,11-;7,13-DiMeC23	309; 112; 266; 182; 196; 210; 168	-	-	-	-	0.08 ± 0.02	-	-
2380	3-MeC23	323; 56; 308	0.02 ± 0.03	-	3.24 ± 0.19	0.04 ± 0.02	0.55 ± 0.13	-	-
2380	C24-ene1	336	-	-	-	-	-	-	0.59 ± 0.25
2386	C24-ene2	336	-	-	-	-	0.09 ± 0.03	-	-
2394	C24-ene3	336	-	-	-	-	0.12 ± 0.04	-	-
2409	n-C24	338	-	-	4.79 ± 0.44	1.88 ± 0.39	3.33 ± 0.49	0.03 ± 0.03	5.92 ± 1.62

2442	10-;12-;14-MeC24	337; 154; 224; 182; 196; 210; 168	-	-	0.11 ± 0.04	0.15 ± 0.11	0.66 ± 0.16	0.1 ± 0.09	1.01 ± 0.37
2463	C25-ene1	350	-	-	-	-	-	-	0.22 ± 0.1
2473	4-MeC24	337; 70; 308	-	-	4.24 ± 1.16	4.11 ± 0.16	2.63 ± 0.36	0.22 ± 0.2	11.79 ± 2.02
2483	C25-ene2	350	-	-	3.41 ± 0.62	-	-	-	7.84 ± 0.82
2483	C25-diene1	348	-	-	-	-	3.93 ± 0.99	-	7.43 ± 1.05
2480	3-MeC24	337; 56; 322	-	-	-	0.31 ± 0.02	-	-	-
2484	C25-ene3	350	-	0.01	-	-	3.84 ± 0.85	-	-
2492	C25-ene4	350	-	-	-	-	1.77 ± 0.36	-	-
2509	n-C25	352	0.32 ± 0.17	1.15 ± 0.96	11.1 ± 0.87	10.67 ± 0.4	9.49 ± 0.56	5.89 ± 5.17	12.91 ± 3.24
2540	C25-diene2	348	-	-	-	-	2.22 ± 0.21	-	-
2542	9-;11-;13-;15-MeC25	351; 140; 252; 168; 224; 196; 224, 168	0.12 ± 0.1	0.13 ± 0.11	3.04 ± 0.89	6.51 ± 2.83	5.26 ± 0.41	11.19 ± 10.08	11.86 ± 1.83
2551	7-MeC25	351; 112; 280	0.03 ± 0.1	-	-	3.18 ± 0.24	1.42 ± 0.46	-	2.39 ± 3.95
2555	C25diene3	348	-	-	6.18 ± 0.93	-	-	-	4.41 ± 1.35
2562	C25diene4	348	-	-	2.48 ± 0.33	-	-	-	-
2557	5-MeC25	351; 84; 308	0.32 ± 0.25	1.12 ± 0.76	-	-	0.39 ± 0.07	-	1.29 ± 0.67
2577	4-MeC25	351; 70; 322	0.21 ± 0.11	6.4 ± 1.81	-	5.69 ± 0.4	2.84 ± 0.93	12.13 ± 1.52	4.29 ± 1.46
2575	C25diene5	348	-	-	4.31 ± 0.22	-	-	-	3.3 ± 1.99
2581	3-MeC25	351; 56; 336	0.19 ± 0.11	6.28 ± 1.96	4.11 ± 0.57	5.81 ± 0.58	3.94 ± 0.64	3.48 ± 2.87	6.44 ± 0.62
2586	5,13-DiMeC25	337; 84; 322; 210; 196	0.11 ± 0.06	-	-	-	-	-	-
2593	C26-ene1	364	-	-	-	-	2.23 ± 0.57	-	-

2600	C26-ene2	364	-	-	-	-	2.52 ± 0.23	-	-
2608	n-C26	366	0.23 ± 0.13	0.82 ± 0.47	-	5.97 ± 0.27	4.51 ± 0.23	2.48 ± 1.75	-
2613	3,7-; 3,9-DiMeC25	337; 56; 350; 126; 280; 154; 252	0.15 ± 0.21	0.33 ± 0.36	-	-	-	-	-
2641	12-; 14; 16-MeC26	365; 182; 224; 210; 196; 238; 168	0.16 ± 0.09	3.21 ± 1.15	-	8.66 ± 0.76	1.89 ± 1.06	2.94 ± 2.17	0.36 ± 0.14
2640	C26diene	362	-	-	-	-	0.53 ± 0.29	-	-
2645	9-MeC26	365; 140; 266	-	-	-	3.03 ± 0.67	-	-	-
2651	7-MeC26	365; 112; 294	-	-	-	0.67 ± 0.1	-	-	-
2651	6-MeC26	365; 98; 308	0.02 ± 0.01	-	-	-	-	-	-
2656	5-MeC26	365; 84; 322	0.05 ± 0.03	0.06 ± 0.03	-	-	-	-	-
2669	4-MeC26	365; 70; 336	0.05 ± 0.03	0.58 ± 0.24	-	5.21 ± 0.16	-	5.08 ± 0.98	0.7 ± 0.29
2681	3-MeC26	365; 56; 350	0.07 ± 0.03	0.3 ± 0.12	-	0.15 ± 0.06	-	0.15 ± 0.04	0.11 ± 0.04
2680	C27-ene1	378	-	-	2.05 ± 0.61	-	-	-	-
2684	C27-ene2	378	-	-	0.6 ± 0.25	-	-	-	-
2689	C27diene1	376	-	-	-	-	1.48 ± 0.36	-	-
2694	C27diene2	376	-	-	-	-	1.95 ± 0.34	-	-
2699	C27-ene3	378	-	-	-	0.11 ± 0.22	2.07 ± 0.36	-	-
2703	C27-ene4	378	-	-	-	0.11 ± 0.22	2.09 ± 1.21	-	-
2711	n-C27	380	3.92 ± 0.41	11.36 ± 3.14	5.13 ± 0.25	8.01 ± 0.45	4.36 ± 0.48	8.05 ± 0.98	0.15 ± 0.05
2744	9-; 11-; 13-; 15-MeC27	379; 140; 280; 168; 252; 196; 224	5.04 ± 0.75	26.96 ± 19.32	-	13.39 ± 0.75	1.01 ± 0.26	20.47 ± 3.31	0.05 ± 0.03
2742	C27diene3	376	-	-	-	-	5.48 ± 0.28	-	-

2752	C28diene1	390	-	-	0.61 ± 0.14	-	-	-	-
2756	C27diene4	376	-	-	0.69 ± 0.32	-	0.48 ± 0.42	-	0.11 ± 0.11
2756	7-MeC27	379; 112; 308	-	-	-	1.26 ± 0.31	-	0.22 ± 0.49	-
2761	5-MeC27	379; 84; 336	3.6 ± 0.47	6.37 ± 1.74	-	-	-	-	-
2760	C27diene5	376	-	-	-	-	0.97 ± 0.26	-	-
2770	11,13-; 11,15-; 11,17-; 13,15-; 13,17-; 15,17-DiMeC27	365; 168; 266; 210; 224; 238; 196	1.67 ± 0.54	0.91 ± 0.48	-	4.3 ± 0.99	-	-	-
2775	4-MeC27	379; 70; 350	-	-	-	-	1.01 ± 0.29	11.18 ± 3.67	-
2775	9,15-: 9.17-DiMeC27	365; 140; 294; 238; 196; 266; 168	-	-	-	5.35 ± 0.77	-	-	-
2779	C27diene6	376	-	-	-	-	2.71 ± 0.15	-	-
2786	3-MeC27	379; 56; 364	3.24 ± 0.31	8.59 ± 1.62	-	0.85 ± 0.49	2.28 ± 0.2	6.76 ± 2.96	-
2784	C28diene2	390	-	-	-	-	0.4 ± 0.08	-	-
2792	5,17-DiMeC27	365; 84; 350; 266; 168	0.58 ± 0.1	7.12 ± 1.3	-	-	-	-	-
2790	5,15-DiMeC27	365; 84; 350; 238; 196	0.12 ± 0.36	1.82 ± 1.06	-	-	0.48 ± 0.37	-	-
2791	5,9-; 5,11-; 5,13-DiMeC27	365; 84; 350; 154; 280; 182; 252; 210; 224	2.53 ± 0.57	-	-	-	-	-	-
2802	C28-ene	392	-	-	-	-	1.01 ± 0.39	-	-
2813	n-C28	394	2.35 ± 1.3	3.3 ± 1.21	-	1.31 ± 0.77	0.28 ± 0.08	0.57 ± 0.43	-
2814	3,9-; 3,11-DiMeC27	365; 56; 378; 154, 280; 282; 252	2.91 ± 0.61	0.06 ± 0.19	-	-	-	-	-
2816	3,7-DiMeC27	365; 56; 378; 126; 308	1.46 ± 0.58	3.8 ± 1.48	-	-	-	-	-
2841	11-; 13-MeC28	393; 168; 266; 196; 238	-	-	-	-	-	1.85 ± 2.3	-
2840	12-; 14-MeC28	393; 182; 252; 210; 224	2.66 ± 0.63	1.41 ± 0.58	-	-	-	-	-

2843	3,9,11-; 3,9,13-; 3,9,15-; 3,9,17-TriMeC27	393; 56; 392; 154; 294; 196; 252; 224; 280; 168	0.88 ± 0.39	-	-	-	-	-	-
2853	6-MeC28	393; 98; 336	0.57 ± 0.22	0.07 ± 0.04	-	-	-	-	-
2858	5-MeC28	393; 84; 350	0.68 ± 0.23	-	-	-	-	-	-
2864	C29diene1	404	-	-	-	-	0.16 ± 0.05	-	-
2868	4-MeC28	393; 70; 364	2.84 ± 0.74	0.27 ± 0.12	-	-	-	0.49 ± 0.48	-
2874	C29-ene1	406	-	2.16 ± 1.06	-	-	-	-	-
2881	C29-ene2	406	1.21 ± 0.79	-	0.84 ± 0.35	-	2.04 ± 2.12	0.07 ± 0.09	-
2880	C29diene2	404	-	0.3 ± 0.14	-	-	-	-	-
2883	C29diene3	404	-	-	-	-	4.09 ± 0.28	-	-
2882	3-MeC28	393; 56; 378	2.27 ± 1.03	-	-	-	-	-	-
2887	5,9-; 5,11-; 5,13-; 5,15-; 5,17-DiMeC28	379; 84; 364; 154; 294; 182; 266; 210; 238	0.48 ± 0.21	-	-	-	-	-	-
2894	C29-ene3	406	-	-	-	-	0.12 ± 0.02	-	-
2897	4,10-; 4,12-; 4,14-; 4,16-; 4,18-; 4,20-DiMeC28	379; 70; 378; 168; 280;196; 252; 224; 308; 140	0.83 ± 0.61	-	-	-	-	-	-
2909	n-C29	408	4.26 ± 0.9	0.51 ± 0.24	1.64 ± 0.36	2.96 ± 1.53	0.34 ± 0.11	0.23 ± 0.17	-
2920	3,7-; 3,9-; 3,11-DiMeC28	479; 56; 392; 126; 322; 154; 294; 182; 266	1.48 ± 0.54	-	-	-	-	-	-
2940	11-; 13-; 15-MeC29	407; 168; 280; 196; 252; 224	7.25 ± 0.51	1.87 ± 0.88	-	-	0.07 ± 0.02	5.96 ± 8.04	-
2953	7-; 9-MeC29	407; 112; 336; 140; 308	3.26 ± 1.16	0.64 ± 0.3	-	-	-	-	-
2964	5-MeC29	407; 84; 364	4.4 ± 4.32	-	-	-	0.02 ± 0.02	-	-
2979	11,17-; 11,19-; 11,21-; 13,17-; 13,19-DiMeC29	393; 168; 294; 266; 196; 322; 140;	2.14 ± 1.19	-	-	-	-	-	-
2983	7,11-DiMeC29	393; 112; 350; 182; 280	1.91 ± 0.85	-	-	-	-	-	-

2985	7,17-DiMeC29	393; 266; 196	-	0.14 ± 0.09	-	-	-	-	-
2986	3-MeC29	407; 56; 392	4.8 ± 0.67	0.08 ± 0.03	-	0.14 ± 0.09	-	0.47 ± 0.47	-
2994	5,9-; 5,11-; 5,13-DiMeC29	393; 84; 378; 154; 308; 182; 280; 210; 252	1.98 ± 0.48	0.1 ± 0.06	0.02 ± 0.04	-	-	-	-
3013	3,7-DiMeC29	393; 56; 406; 126; 336	-	0.45 ± 0.23	-	-	-	-	-
3019	3,9-; 3,11-DiMeC29	393; 56; 406; 154; 308; 182; 280	6.44 ± 0.76	-	0.03 ± 0.07	-	-	-	-
3031	3,7-DiMeC29 (peak shifted in Bg)	393; 56; 406; 126; 336	0.63 ± 0.31	-	-	-	-	-	-
3041	3,7,11-; 3,7,13-; 3,7,15-; 3,7,17-; 3,9,11-; 3,9,13-; 3,9,15-; 3,9,17-TriMeC29	421; 56; 420; 126; 350; 196; 280; 224; 252; 280; 196; 154; 322;	4.4 ± 0.68	-	-	-	-	-	-
3061	14,18-; 14-22-DiMeC30	407; 210; 266; 280; 196; 336; 140;	0.86 ± 0.43	-	-	-	-	-	-
3063	4-MeC30	421; 70; 392	0.34 ± 0.24	-	-	-	-	-	-
3075	C31ene	434	0.18 ± 0.11	0.01 ± 0.02	-	-	-	-	-
3076	C31diene	432	-	-	-	-	0.25 ± 0.13	-	-
3080	5,9-; 5,11-; 5,13-; 5,15-DiMeC30	407; 84; 392; 154; 322; 182; 294; 210; 266; 238	0.17 ± 0.09	0.03 ± 0.1	-	-	-	-	-
3088	4,8-; 4,10-; 4,12-DiMeC30	407; 70; 406; 140; 336; 168; 308; 196; 280	2.39 ± 0.86	0.01 ± 0.04	-	-	-	-	-
3102	3,9-; 3,11-DiMeC30	407; 56; 420; 154; 322; 182; 294	0.59 ± 0.41	-	-	-	-	-	-
3112	4,8,12-; 4,8,14-; 4,8,16-TriMeC30	435; 70; 420; 140, 350; 210; 280; 238, 252; 266; 224	0.16 ± 0.12	-	-	-	-	-	-
3130	11-; 13-; 15-MeC31	435; 168; 308; 196; 280; 224; 252	3.13 ± 0.76	-	-	-	-	-	-
3154	13,17-; 15,17-DiMeC31	421; 196; 294; 266; 224	2.16 ± 1	0.11 ± 0.38	-	-	-	-	-
3178	5,9-;5,11-; 5,13-; 5,15-;5,17-DiMeC31	421; 84; 406; 154; 336; 182; 308; 210; 280; 238; 252; 266; 224	1.83 ± 0.66	-	-	-	-	-	-
3203	3,9-; 3,11-; 3,13-; 3,15-DiMeC31	421; 56; 434; 154; 336; 182; 308; 210; 280; 238; 252	0.52 ± 0.25	0.01 ± 0.03	0.01 ± 0.03	-	0.02 ± 0.02	-	-

3477	C35-ene	490	-	-	-	-	0.12 ± 0.04	-	-
3531	11-MeC35	491; 168; 364	-	-	-	-	-	-	0.55 ± 0.31
3629	12-MeC36	505; 182; 364	-	-	-	-	-	-	0.09 ± 0.06
3729	11-; 13-MeC37	519; 168; 392; 196; 364	-	-	0.05 ± 0.05	-	-	-	2.28 ± 1.11
3751	11,15-DiMeC37	505; 168; 406; 238; 336	-	-	-	-	-	-	0.45 ± 0.23
3774	5,15-; 5,17-DiMeC37	505; 84; 490; 236; 336; 266; 308	-	-	-	-	-	-	0.65 ± 0.33
3840	C39diene	544	-	-	0.28 ± 0.1	-	-	-	-
3876	C39-ene	546	-	-	2.24 ± 0.18	-	-	-	-
3927	11-;13-;15-MeC39	547; 168; 420; 168; 392; 224; 364	-	-	2.74 ± 0.41	-	0.06 ± 0.02	-	0.8 ± 0.37
3949	11,15-DiMeC39	533; 168; 434; 238; 364	-	-	-	-	-	-	0.57 ± 0.26
3953	13,17-DiMeC39	533; 196; 406; 266; 336	-	-	0.24 ± 0.06	-	-	-	-
3973	5,15-; 5,17-DiMeC39	533; 84, 518; 238; 364; 266; 336	-	-	-	-	-	-	0.32 ± 0.16
4054	C41diene	572	-	-	5.32 ± 0.18	-	-	-	-
4074	C41-ene	574	-	-	1.92 ± 0.5	-	-	-	-
4084	C41-ene	574	-	-	2.49 ± 0.36	-	-	-	-
4143	11-; 13-; 15-; 17-MeC41	575; 168; 448; 196; 420; 224, 392; 252; 364	1.72 ± 1.2	0.52 ± 0.37	0.62 ± 0.23	-	0.11 ± 0.03	-	0.02 ± 0.04
4173	13,15-; 13,17-DiMeC41	561; 196; 434; 238; 392; 266; 364	0.99 ± 1.52	0.6 ± 1.98	0.58 ± 0.15	-	-	-	-
