# Supplementary figures and images for: Neglected Very Long-Chain Hydrocarbons and the Incorporation of Body Surface Area Metrics Reveal Novel Perspectives for Cuticular Profile Analysis in Insects
Source: Insects. 2022 Jan 12;13(1):83. doi: 10.3390/insects13010083 (PMC8778109; doi:10.3390/insects13010083)

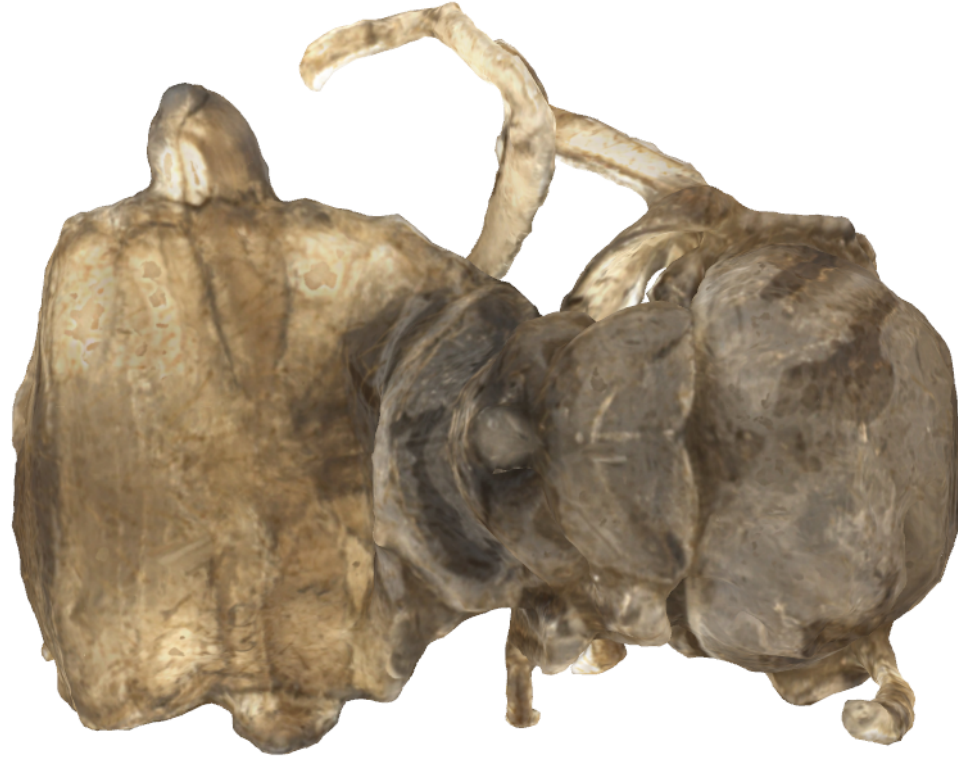

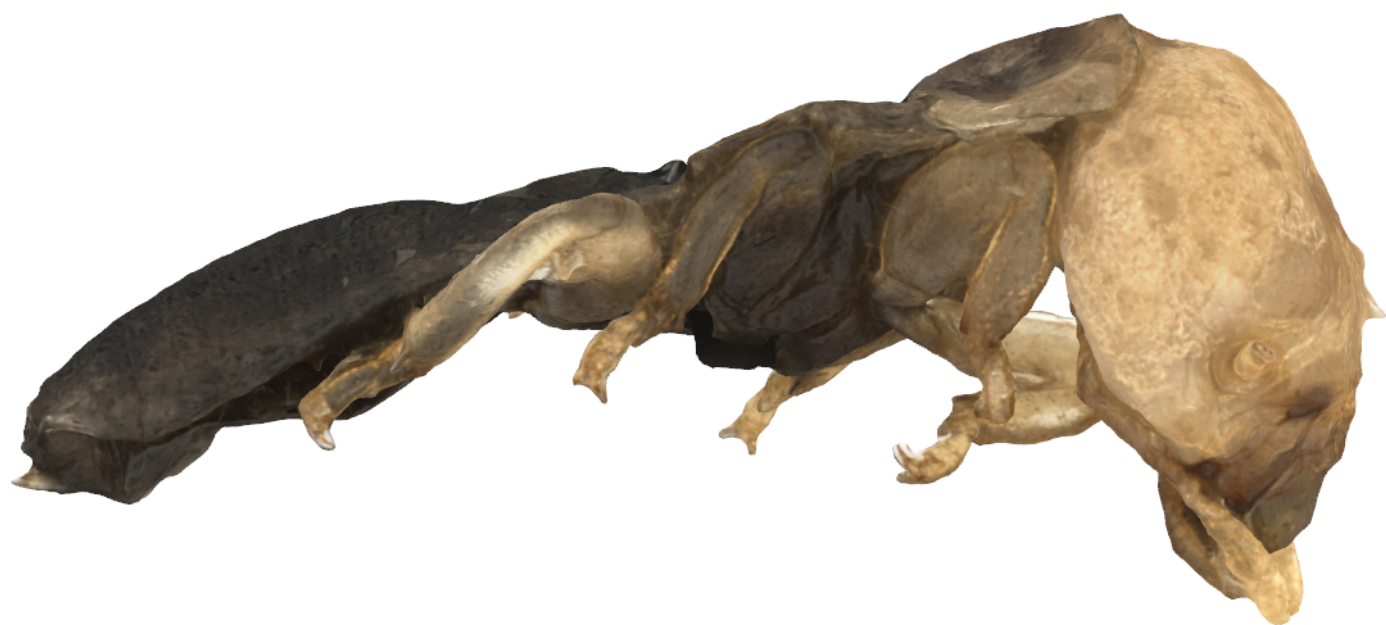

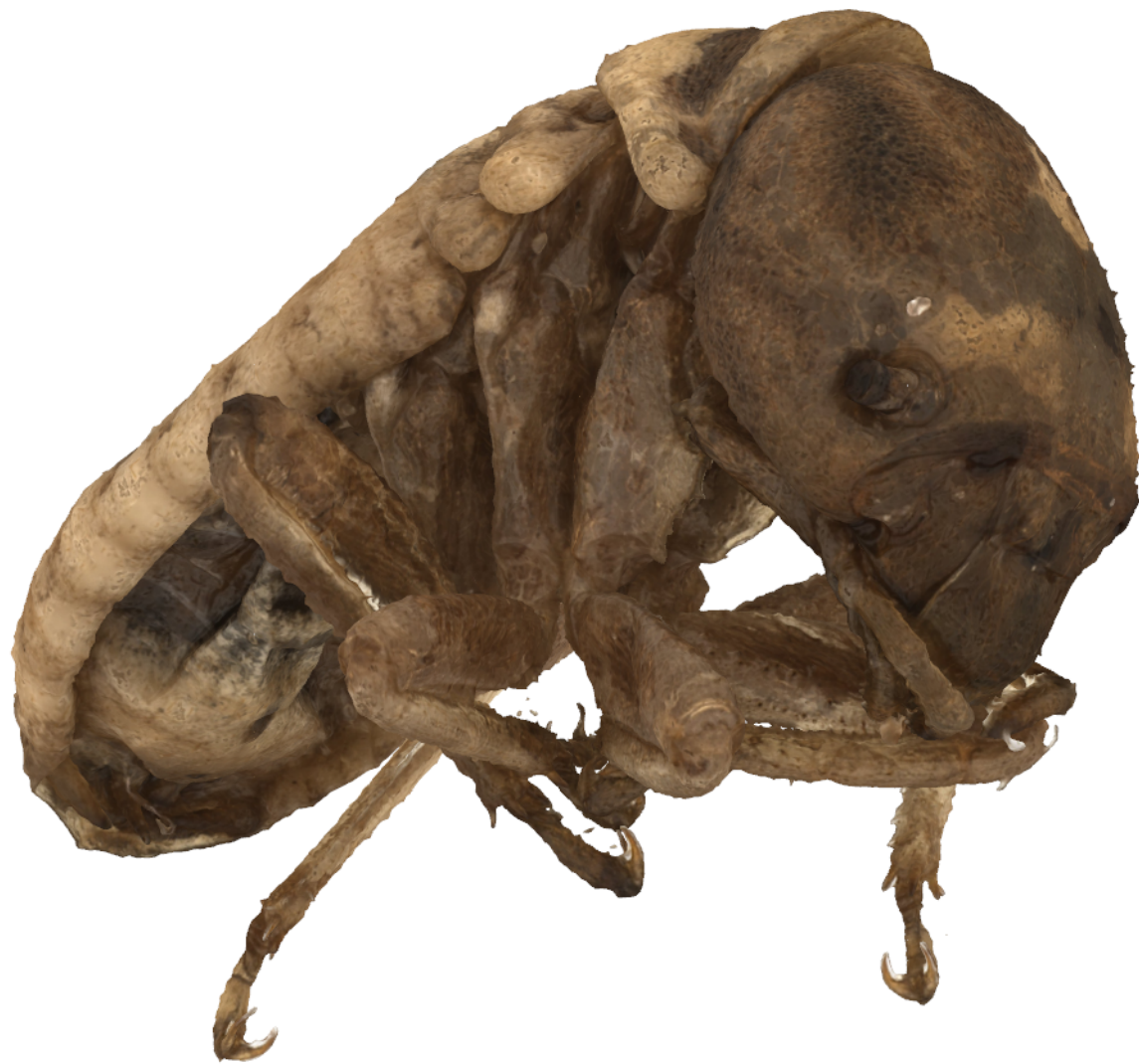

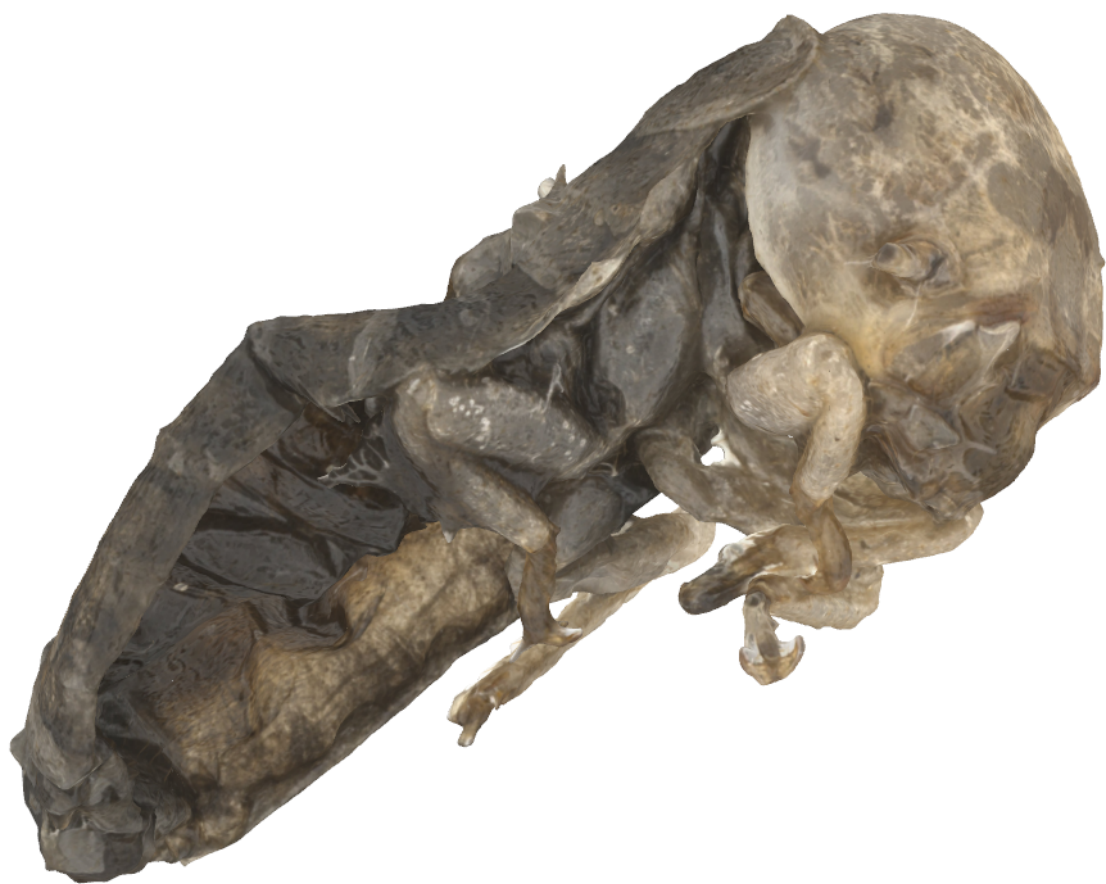

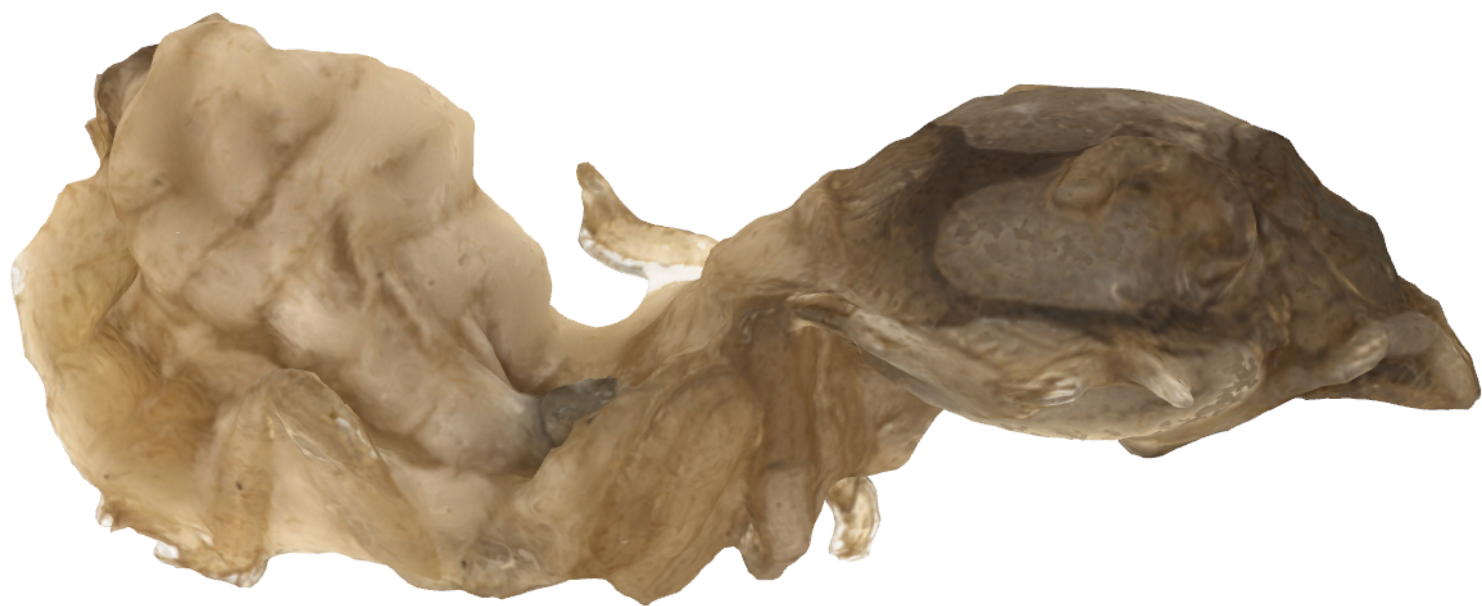

Supplement: Supplementary file 1 [file insects-13-00083-s001.zip › Fig-S1.pdf]

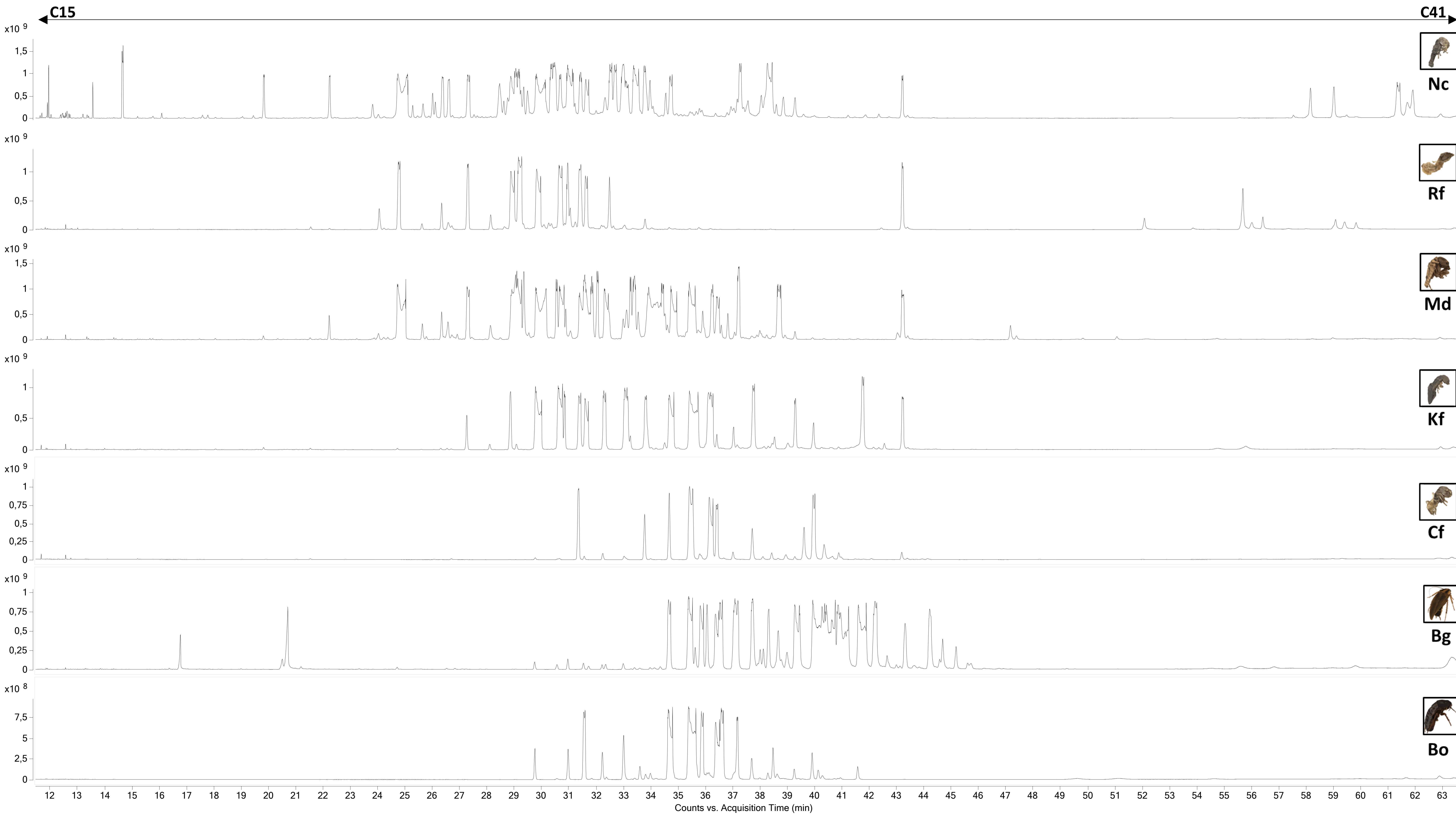

Supplement: Supplementary file 1 [file insects-13-00083-s001.zip › Fig-S3.pdf]
